# Obscurin variants and inherited cardiomyopathies

**DOI:** 10.1007/s12551-017-0264-8

**Published:** 2017-05-05

**Authors:** Steven Marston

**Affiliations:** Imperial College London, London, UK

**Keywords:** Cardiac muscle, Obscurin, Mutation, Dilated carriomyopathy, Hypertrophic cardiomyopathy

## Abstract

The inherited cardiomyopathies, hypertrophic cardiomyopathy (HCM), dilated cardiomyopathy (DCM) and left ventricular non-compaction (LVNC), have been frequently associated with mutations in sarcomeric proteins. In recent years, advances in DNA sequencing technology has allowed the study of the giant proteins of the sarcomere, such as titin and nebulin. Obscurin has been somewhat neglected in these studies, largely because its functional role is far from clear, although there was an isolated report in 2007 of obscurin mutations associated with HCM. Recently, whole exome sequencing methodology (WES) has been used to address mutations in OBSCN, the gene for obscurin, and OBSCN variants were found to be relatively common in inherited cardiomyopathies. In different studies, 5 OBSCN unique variants have been found in a group of 30 end-stage failing hearts, 6 OBSCN unique variants in 74 HCM cases and 3 OBSCN unique variants in 10 LVNC patients. As yet, the number of known potentially disease-causing OBSCN variants is quite small. The reason for this is that mutations in the OBSCN gene have not been recognised as potentially disease-causing until recently, and were not included in large-scale genetic surveys. OBSCN mutations may be causative of HCM, DCM and LVNC and other cardiomyopathies, or they may work in concert with other variants in the same or other genes to initiate the pathology. Currently, the function of obscurin is not well understood, but we anticipate that many more OBSCN variants linked to cardiomyopathy will be found when the large cohorts of patient sequences available are tested. It is to be hoped that the establishment of the importance of obscurin in pathology will stimulate a thorough investigation of obscurin function.

The inherited cardiomyopathies, particularly hypertrophic cardiomyopathy (HCM) dilated cardiomyopathy (DCM) and left ventricular non-compaction (LVNC), have been frequently associated with mutations in the sarcomeric proteins. In recent years, advances in DNA sequencing technology have allowed the study of the giant proteins of the sarcomere, notably truncating mutations in the titin gene (TTNtv), which have been found to be responsible for up to 25% of familial DCM cases (Roberts et al. 2015), and mutations in nebulin (NEB), the most common mutations causing skeletal muscle myopathies such as nemaline myopathy (Lehtokari et al. 2014).

Obscurin has been somewhat neglected in these studies, largely because its functional role is far from clear. There was an isolated report in 2007 of obscurin mutations associated with HCM but this was not followed up for several years. Recently, whole exome sequencing methodology (WES) has been used to address mutations in OBSCN, the gene for obscurin, and OBSCN variants were found to be relatively common in inherited cardiomyopathies. Marston et al.’s (2015) study found 5 OBSCN unique variants in 4 individual hearts from a group of 30 end-stage failing hearts, Xu et al. (2015) found 6 OBSCN unique variants in 74 HCM cases and Rowland et al. (2016) found 4 OBSCN unique variants in 335 DCM and LVNC patients, of which OBSCN variants were associated with 3 out of 11 LVNC cases (Table 1).

**Table 1:** Cardiomyopathy-linked Obscurin mutations based on the Obscurin B sequence NP_001092093 (Fukuzawa et al. 2005)

	Mutation	Pathology	Domain
Arimura et al.	R4344Q	HCM	Ob58
	A4484T	HCM	Ob59
Marston et al.	E963K	DCM	Ob 9
	V2161D	DCM	Ob 21
	F2809 V	DCM	Ob 27
	R4856H	DCM	Ob 47
	D5966N	DCM	PH
Rowland et al.	T6309R	LVNC	Between Ob66 and Ob67
	S6990P	LVNC	Between kinase I and Ob69
	A6993P	DCM	Between kinase I and Ob69
	c25367-1G > C	LVNC	
Xu et al.	A996fs	HCM	Ob 10
	A1088fs	HCM	Ob 11
	A1272fs	HCM	Ob13
	A1640fs	HCM	Ob 17
	G7500R	HCM	Ob 69

The locations of the mutations are shown in the obscurin B sequence in Fig. 1. Note that the published amino acid numbers in Rowland et al. 2016 were based on NP_001258152 and have been converted to NP_001092093 numbering for comparison

On this basis, potentially disease-causing OBSCN variants seem to be fairly common in all three diseases. As yet, the number of known potentially disease-causing OBSCN variants is quite small. The reason for this is that mutations in the OBSCN gene have not been recognised as potentially disease-causing until recently, and are not included in current large-scale genetic surveys, For instance, Alfares et al. (2015) studied 2912 HCM cases, Ware et al. (2016) studied 172 peripartum cardiomyopathy cases and Haas et al. (2014) studied 639 DCM cases. It is noteworthy that the largest study to date, in which 46 genes in 7855 cardiomyopathy cases were compared with 60,706 reference samples, did not analyse variants in the OBSCN gene (Walsh et al. 2017). We anticipate that, when these large cohorts are re-examined for OBSCN disease-related variants, a considerable number will be found.

All the recent studies on OBSCN variants have involved whole exon sequencing with the unique variants being identified by comparison with large reference databases, such as Exacs and EVS, and their pathogenicity predicted using algorithms such as SIFT. This approach, based on small populations, may be biased in patient ethnicity and other factors. It is recognised that such conclusions may give a false positive association of disease with variants especially in a large protein (Walsh et al. 2017). Variants that cause chain termination, as found in the Rowland et al. (2016) and Xu et al. (2015) studies, are more likely to be pathogenic than missense mutations; however, none of the current studies on OBSCN variants have any family history showing the variant segregating with the disease, which is required to confirm causality. In the absence of additional data, we cannot exclude the possibility that the observed variants are simply bystander effects and that the true disease-causing mutation is elsewhere.

The only study that has directly investigated the obscurin molecule in heart muscle with identified mutations was Marston et al.’s (2015) study of familial DCM. where obscurin haploinsufficiency was demonstrated for all the OBSCN variant samples compared with fDCM with mutations identified in other genes or in donor heart samples. This, therefore, increases the confidence that OBSCN variants and familial DCM are linked in this particular group of samples from patients who had transplants for end-stage heart failure. In their studies on TTNtv mutations, Roberts et al. (2015) found a strong correlation of end-stage failure with mutation; however, the other studies on OBSCN used larger groups with less severe disease and the association is less certain.

OBSCN variants may be monogenic causes of cardiomyopathy or they may contribute to the disease phenotype in concert with other variants. It is therefore relevant to note that a significant proportion of the reported OBSCN variant samples also had a second disease-related variant. The HCM patient studied by Arimura et al. (2007) had two OBSCN mutations: R4344Q and A4484T. Arimura suggested that only the R4344Q variant was pathogenic, and it has since been shown that the A4484T variant is, in fact, common (15%) in black Americans. The A4484T variant has recently been downgraded to “disease-causing?” in a recent analysis of pathogenicity of putative HCM mutations (Manrai et al. 2016); however, there is no direct evidence for the relative roles of these variants and both may be required for pathogenicity. Similarly, Marston et al. (2015) found a DCM sample with two OBSCN mutations: V2161D and F2809V. This paper also lists two other double variants: OBSCN E963K + DSP R1537C and OBSCN R4856H + SCN5A S216L; both the DSP and the SCN5A variant have been previously noted as being associated with cardiomyopathies but not causative (Marangoni et al. 2011; Xu et al. 2010). On balance, it seems likely that most OBSCN variants contribute to cardiomyopathy rather than being a monogenic cause.

With other disease-causing mutations such as MyBP-C and nebulin, the discovery of disease-related mutations has stimulated investigation of the structure and function of the protein in muscle, but this has not happened for obscurin. The structure of obscurin is well established based on the amino acid sequence derived from cDNA sequencing (Fig. 1). The canonical sequence of obscurin B, the largest isoform, has 7968 amino acids. It is a typical modular protein being largely made up from multiple Ig domains plus three Fn3 domains, one IQ domain, one DH domain and one PH domain (Fukuzawa et al. 2005). In muscle, two large alternatively spliced isoforms are found, obscurin A and B. Obscurin A contains a C-terminal interaction site with sAnk1 and other ankryn isoforms, whilst obscurin B has two C-terminal kinase domains. Three of the OBSCN variants were found to be in the two-kinase domain sequence specific to obscurin B and none have been found in the obscurin A C-terminal sequence (Table 1). Western blots of obscurin in human heart muscle show a single band with an apparent molecular mass of 960 kD, suggesting that obscurin B is the main isoform in human heart (Marston et al. 2015), although both isoforms are present in rat and mouse hearts. It should be recognised that there are further isoforms of obscurin A and B expressed in many non-muscle tissues with shorter N-terminal sequences and a common C-terminus (Ackermann et al. 2014). OBSCN variants have been associated with pathologies including Wilm’s tumour and with aspirin sensitivity in asthmatics which could involve these smaller isoforms (Perry et al. 2013).

**Fig. 1:**
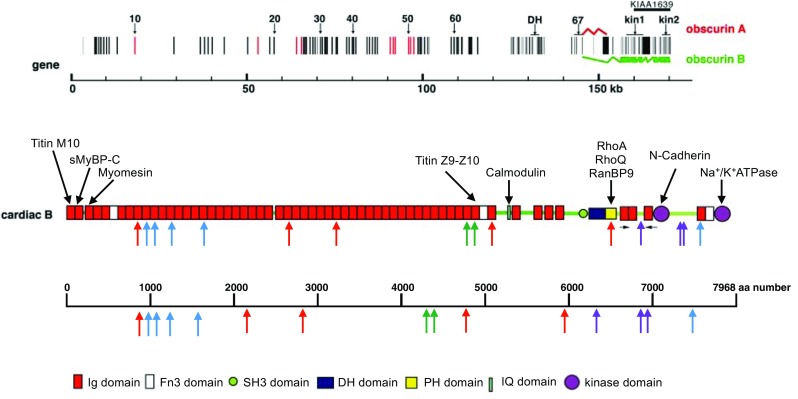
Obscurin structure and cardiomyopathy-associated mutations, based on the model of Fukuzawa et al. (2005). *Top* gene structure, showing the exons and alternative splicing that yields obscurin A or B. *Middle* schematic domain structure of obscurin B. The proposed interaction sites are shown above the model and the location of the mutations are shown below (refer to Table 1). Note that this model is not to scale. *Bottom* location of the mutations in the 7688 amino acid sequence of obscurin B (NP_001092093)

Obscurin has been found to interact with numerous other proteins in vitro. Best characterised are the N-terminal domains that interact with M-line titin and myomesin which anchor the N-terminus of obscurin to the M-line complex and contribute to its stability. Titin M10 Ig domain interacts with the N-terminal Ig domain of obscurin and the myomesin Ig domain 4 interacts with the third Ig domain of obscurin (Gautel 2011; Pernigo et al. 2010, 2017). In addition, obscurin domains Ob58/59 interact with titin Ig domains Z9/Z10 in vitro, potentially anchoring obscurin to the Z-disk (Bang et al. 2001).

The sAnk1 binding site in the C-terminus of obscurinA has also been extensively studied. This interaction is particularly significant because it represents a direct molecular link between the contractile sarcomere and the SR membrane, illuminating a possible mechanism for the alignment and anchoring of the SR around the developing myofibril and during contraction (Bagnato et al. 2003; Kontrogianni-Konstantopoulos et al. 2003; Perry et al. 2013). The PH domain interacts with Rho proteins. It can specifically induce exchange of GDP for GTP in the small GTPases, RhoA and RhoQ (Ford-Speelman et al. 2009; Perry et al. 2013; Russell et al. 2002), leading to activation of their downstream effectors. Since RhoA activity plays an important role in normal myofibril growth and during pathologic hypertrophy, this interaction could be physiologically significant. The kinase domains of obscurin appear to be active and have been shown to phosphorylate N-cadherin and Na^+^/K^+^ ATPase (Hu and Kontrogianni-Konstantopoulos 2013; Russell et al. 2002). In striated muscles, obscurin has been shown to interact with a novel isoform of sleletal MyBP-C (Ackermann et al. 2009) . The functional relevance of these domain interactions is still not well understood, but the current consensus is that obscurin is involved in the development of the myofibril and sarcoplasmic reticulum. Obscurin also forms a physical link between the M-line and the sarcoplasmic reticulum or sarcolemma which could include a mechanosensing function.

The OBSCN variants found so far seem to be distributed throughout the molecule (Fig. 1), mostly with no apparent connection to any functional domain. Arimura et al. (2007) have proposed that the R4344Q mutation affects binding of obscurin to Z-line titin (domains Z9-Z10), and Marston et al. (2015) noted obscurin haploinsufficiency but did not measure any functional properties. The best clues to the physiological function of obscurin are provided by the obscurin knockout mouse model (Lange et al. 2009); however, the effects of knockout have only been fully studied in skeletal muscle rather than in cardiac muscle and no cardiac defect was reported. In skeletal muscle, absence of obscurin results in disorganisation of microtubules at the sarcolemma and delocalisation of dystrophin at costameres. More striking abnormalities appear with intense exercise, including fibre damage, atrophy and degeneration which revert when exercise is stopped (Randazzo et al. 2017). It was proposed that the absence of obscurin causes the failure of the muscle sarcolemma to support the mechanical stress of intensive chronic contraction. In the heart, this may be manifested as development of a hypo-contractile heart under stress which could trigger DCM.

In conclusion, despite limited investigations, mutations in the OBSCN gene are emerging as significant in a range of cardiomyopathies. OBSCN mutations may be causative of HCM, DCM and LVNC and other cardiomyopathies, or they may work in concert with other variants in the same or other genes to initiate the pathology. Currently, the function of obscurin is not well understood other than the suggestion that it is not an essential gene and that its functions involve stabilisation of sarcomere to sarcoplasmic-reticulum and sarcomlemma links that may only become important during high levels of contraction. We anticipate that many more OBSCN variants linked to cardiomyopathy will be found when the large cohorts of patient sequences available are tested. It is to be hoped that the establishment of the importance of obscurin in pathology will stimulate a thorough investigation of obscurin function.
